# Effectiveness of interventions during NICU hospitalization on the neurodevelopment of preterm infants: a systematic review protocol

**DOI:** 10.1186/s13643-017-0613-5

**Published:** 2017-11-03

**Authors:** Marilyn Aita, Robyn Stremler, Nancy Feeley, Andréane Lavallée, Gwenaëlle De Clifford-Faugère

**Affiliations:** 1 Faculty of Nursing, Université de Montréal, Montréal, Canada; 2 Research Center of the CHU Sainte-Justine, Montréal, Canada; 3 Lawrence S. Bloomberg Faculty of Nursing, University of Toronto, Toronto, Canada; 4 Hospital for Sick Children (SickKids), Toronto, Canada; 5 Ingram School of Nursing, McGill University, Montréal, Canada; 6 Centre for Nursing Research and Lady Davis, Jewish General Hospital, Montréal, Canada

**Keywords:** Preterm infants, Neurodevelopment, NICU, Interventions, Hospitalization

## Abstract

**Background:**

Previous systematic reviews have examined preterm infants’ long-term neurodevelopment after neonatal intensive care unit (NICU) discharge, although none have explored the effectiveness of interventions on preterm infants’ neurodevelopment during NICU hospitalization. The aim of this review is to evaluate whether interventions, i.e., sensory stimulation, parental involvement, and control of environment, improve preterm infants’ neurodevelopment during their NICU hospitalization.

**Methods:**

Experimental studies such as randomized controlled/clinical trials (RCTs) and cluster RCT will be included in this systematic review. Selected studies will be published in English or in French, in the past 15 years from 2002 to 2017. The following electronic databases will be searched to locate relevant studies: CINAHL, MEDLINE, PubMed, EMBASE (OVID), Cochrane Database of Systematic Reviews, Cochrane Central Register of Controlled Trials (CENTRAL), and Web of Science. For all steps in selecting studies, agreement will be reached between two experts in neonatology. Data extraction will be performed independently by the two same experts and will then be compared. The Cochrane assessment tool will be used to screen the studies for risk of bias. A meta-analysis will be performed if the included studies are sufficiently homogeneous. Results will be analyzed using a standardized mean difference (with a 95% confidence interval). Statistical heterogeneity will be evaluated using the *χ* ^2^ test at the significance level of 0.1 and the *I* ^2^ with the classification suggested by PRISMA-P. If possible, subgroup analyses will be carried out considering preterm infants’ gestational age, length of NICU hospitalization, and the characteristics of the intervention such as who delivered it, the type, the dose, the frequency, and the duration. Data synthesis will be performed using the RevMan 5.1 software. Publication bias and selection of variables in publication will be examined using the graphical method of funnel plot and with the statistical test of Egger. Quality of the evidence of all outcomes will be assessed using the Grades of Recommendations Assessment, Development and Evaluation (GRADE) Working Group.

**Discussion:**

The results of this systematic review will highlight which interventions are effective for promoting preterm infants’ neurodevelopment during NICU hospitalization and will contribute to the body of knowledge in neonatal care by providing guidance for NICU clinical practice and research.

**Systematic review registration:**

PROSPERO CRD42017047072

**Electronic supplementary material:**

The online version of this article (10.1186/s13643-017-0613-5) contains supplementary material, which is available to authorized users.

## Background

Because of an interruption of intrauterine growth and a neonatal intensive care unit (NICU) hospitalization, preterm infants’ are more susceptible to encounter significant short- and long-term neurodevelopmental impairments. In fact, a recent epidemiological study reveals that over 25% of infants born between 28 and 32 weeks of gestation have neurodevelopmental disabilities such as cognitive, motor, visual, or hearing deficits, at the age of 2 years, and this proportion increases by 15% at age ten to reach around 40% [1].

The preterm infants’ neurodevelopment is influenced during the NICU hospitalization where the experiences they encounter can have significant consequences for the development and functioning of their brain [2, 3, 4]. While hospitalized in the NICU, the infant’s brain goes through a period of critical growth between 24 and 40 weeks of gestation [4, 5]. A series of complex neurological events occur, such as the establishment of synaptic and neuronal connections, as well as the proliferation of significant structures like the thalamus, the cortex, and the cerebellum, all vulnerable to exogenous and endogenous experiences [4]. There is consensus that NICU-related factors which influence the preterm infants’ neurodevelopment during hospitalization are, among others, environmental stimulation [6–11], interactions between parents and infants [10–12], and the caregiving experience [5]. Hence, interventions aimed at sustaining or modifying these NICU factors during hospitalization should promote preterm infants’ optimal neurodevelopment.

The effectiveness of interventions initiated during the NICU hospitalization has been evaluated in previous systematic reviews on preterm infants’ health outcomes. Those interventions include developmental care [13], NICU noise reduction [14], skin-to-skin contact [15], and early interventions involving parents [16]. Although these reviews have all included preterm infants’ long-term neurodevelopment after discharge (i.e., from 12 to 24 months of age) as an outcome, none specifically examined the effectiveness of such interventions on the preterm infants’ neurodevelopment during their NICU hospitalization. Identifying effective interventions promoting preterm infants’ neurodevelopment as soon as they are hospitalized in the NICU will guide clinical neonatal clinical care and may foster preterm infants’ long-term development. Moreover, the results of this systematic review will provide a better understanding of the effectiveness of early interventions on the underlying processes of preterm infants’ neurodevelopment during hospitalization in addition to guiding neonatal research.

## Objective

The objective of this systematic review is to evaluate whether interventions implemented in the NICU improve preterm infants’ neurodevelopment during their hospitalization. This systematic review is guided by the following question: During NICU hospitalization, what is the effectiveness of interventions on the neurodevelopment of preterm infants receiving these interventions compared to those who did not receive these interventions?

## Methods

The protocol for this systematic review was developed according to the Preferred Reporting Items of Systematic Reviews and Meta-Analysis for Protocols (PRISMA-P) 2015 [17]. The PRISMA checklist is provided as an additional file to the protocol (see Additional file 1). The systematic review protocol has been registered in the International Prospective Register of Systematic Reviews (PROSPERO): CRD42017047072.

### Eligibility criteria of the selected studies

The studies selected in this systematic review will meet the following eligibility criteria which are described according to the study design (including publication, language, and year) participants, interventions, comparators, and outcomes.

#### Study design

Experimental studies such as randomized controlled/clinical trials (RCTs) and cluster RCTS will be included. We will exclude studies where the randomization process was not random, i.e., assigning alternatively participants, first half of participants to one group or first NICU receiving the intervention compared to a second one not receiving the intervention or assignment of participants by characteristic such as date of birth. We will also exclude quasi-experimental studies (no randomization or no comparative group), as well as all descriptive and case-control studies. Only including RCTs in this systematic review is justified by the fact that this research design provides the highest level of evidence**.** Accordingly, strongest inferences can be concluded from systematic reviews which include RCTs with reliable findings [18]. Selected studies will be written in English or in French and will have been published in the past 15 years, from 2002 to 2017.

#### Participants

Studies including preterm infants, born between 24 to 36 6/7 weeks of gestation will be considered. Infants with major brain abnormalities or any other health situation influencing neurodevelopment, such as intra-ventricular hemorrhage greater than grade II or brain malformation, will be excluded.

#### Interventions

All interventions, aimed either at preterm infants or parents, instigated during NICU hospitalization will be considered. These interventions can be delivered either by healthcare professionals or parents or by both. For example, those may include either sensory stimulation, parental involvement, control of environment. Multi-faceted interventions will be excluded from this review as it will be difficult to estimate which component was more efficacious in promoting infants’ neurodevelopment and will preclude meta-analysis if applicable. If indicated, we will contact authors to obtain details about the interventions.

#### Comparator

All types of comparator groups, such as non-exposed control group or a group exposed to different intervention, will be included in this systematic review.

#### Outcome

Studies reporting preterm infants’ neurodevelopment as an outcome evaluated with a standardized instrument, scale, or test during the NICU hospitalization will be included in this systematic review. Examples of standardized instruments, scales, and tests measuring neurodevelopment are the Assessment of the Preterm Infants Behaviors (APIB) [19], NICU Network Neurobehavioral Scale [20], General Assessment Movement (GMA) [21], and electroencephalography (EEG).

### Search strategy

An expert librarian will be consulted to create a list of keywords to be used individually or in combination to conduct the search. The following electronic databases will be searched with MeSH/Thesaurus terms to screen for relevant studies for this systematic review: CINAHL, MEDLINE, PubMed, EMBASE (OVID), Cochrane Database of Systematic Reviews, Cochrane Central Register of Controlled Trials (CENTRAL), and Web of Science (see Additional file 2 for an example of a search strategy in MEDLINE). The Scopus database will also be reviewed to search for trials in conference proceedings or in Proquest for thesis and dissertations. We will also look at the clinicaltrials.gov website to identify trials which are presently underway. The reference list of studies identified for this review will be screened to identify other potential studies.

### Screening of studies

All references of studies selected for this review will be managed in the EndNote©. All duplicates of studies will be identified by two separate reviewers who are experts in neonatology and then compared to ensure appropriate deletion of studies. These two reviewers will then screen the remaining studies for eligibility in two steps. The first step will consist of reviewing all studies’ titles and abstracts to identify which studies meet the eligibility criteria. The second step will consist of reviewing the studies’ full-text reports to evaluate their appropriateness to be included in the systematic review. For all steps, i.e., deleting duplicates and reviewing titles/abstracts and then full-text papers, an agreement will be reached between the two reviewers. In case of disagreements at any of these steps, a third expert will be consulted. Numbers of studies reviewed, included, and excluded and reasons for exclusion will be noted in a report form.

### Data extraction

Data extraction will be performed independently by two experts and will then be compared. A pilot of data extraction will be done with ten studies. The extracted information will be recorded including the study title, the trial number, the authors and publication year, the country of publication, the research design, the sample size, the description of the intervention (name, who delivered it, comparator, dose, frequency, timing, duration, follow-up), sample (gestational age, age at assessment, weight at birth, gender, severity of illness score), timing of assessment, and outcome, including the definition of neurodevelopment as well as the instruments, scales, or tests used to measure this outcome. Multiples reports of the same study will only be included if data reported are different in each report. All extracted data will be recorded in RevMan 5.1.

## Assessment of risk of bias

The Cochrane’s risks of bias [22] assessment tool will be used to screen the studies’ risk of bias which will be classified as low, high, or unclear risk of bias. The tool screens for sequence generation, allocation concealment, blinding, incomplete outcome data, and selective outcome reporting. Risk of bias will be assessed independently by two reviewers. A third expert will be involved if a disagreement cannot be solved among the two other experts.

## Data synthesis

A meta-analysis will be performed if the included studies are sufficiently homogeneous.

### Measuring treatment effect

The results will be analyzed using a standardized mean difference (with a 95% confidence interval) since different tools measuring preterm infants’ neurodevelopment will be considered in this review.

### Missing data

Authors of studies will be contacted to obtain missing data; if data cannot be recovered, then a method of permutation will be used.

### Assessment of heterogeneity

The heterogeneity of the studies will be evaluated using the *χ* ^2^ test at the significance level of 0.1 and the *I* ^2^ with the classification suggested by the PRISMA-P: 0 to 40% not important heterogeneity, 30 to 60% moderate heterogeneity, 50 to 90% considerable heterogeneity, and 75% 100% significant heterogeneity [17]. Considerable heterogeneity > 50% will be explored using subgroup analysis.

### Subgroup analysis

If possible, subgroup analysis will be carried out considering the preterm infants’ gestational age (GA) at birth and at assessment, the length of hospitalization, the characteristics of the intervention such as who delivered it, the type, dose, frequency, and duration.

### Data synthesis

Data synthesis will be performed using the RevMan 5.1 software. If the heterogeneity of the studies is not significant, a fixed effect with the Mantel-Haenszel method [23] will be chosen for data synthesis; if the studies’ heterogeneity is considerable (i.e., *χ* ^2^, *p* > 0.1 and *I* ^2^ ≥ 50% [17]), the data synthesis will only be narrative.

### Meta-analysis of bias

Publication bias and selection of variables in publication will be examined using the graphical method of funnel plot if > 10 studies are included in this review and with the statistical test of Egger [24]. In the presence of bias, even small, a random estimation will be necessarily selected for data synthesis.

## Quality of evidence

The quality of the evidence for all outcomes will be assessed using the Grades of Recommendations Assessment, Development and Evaluation (GRADE) Working Group, which is considered a valid approach [25].

## Discussion

The results of this systematic review will highlight which interventions are effective for promoting preterm infants’ neurodevelopment during NICU hospitalization and will guide neonatal clinical care accordingly. Implementing effective interventions as soon as preterm infants are in the NICU will promptly optimize their neurodevelopment and may prevent further long-term neurological impairments in this vulnerable population. Findings of this systematic review will also highlight the state of knowledge on interventions which may only be effective during NICU hospitalization and will guide neonatal research with recommendations. Findings of this review will be published in a scientific journal and will contribute to the body of knowledge in neonatal care by providing guidance for the NICU clinical practice and neonatal research.

## Additional files

Additional file 1: PRISMA-P 2015 Checklist. (PDF 249 kb)

Additional file 2: MEDLINE search strategy. (PDF 181 kb)

## Abbreviations

GA: Gestational age
NICU: Neonatal intensive care unit
